# A Scoping Review of Interventions to Supplement Spoken Communication for Children with Limited Speech or Language Skills

**DOI:** 10.1371/journal.pone.0090744

**Published:** 2014-03-13

**Authors:** Maria Antonella Costantino, Maurizio Bonati

**Affiliations:** 1 Child and Adolescent Neuropsychiatric Unit, IRCCS Foundation Ca’ Granda, Ospedale Maggiore Policlinico, Milan, Italy; 2 Laboratory for Mother and Child Health, Department of Public Health, IRCCS - “Mario Negri” Pharmacological Research Institute, Milan, Italy; Stony Brook University, United States of America

## Abstract

**Background:**

Augmentative and Alternative Communication (AAC) is used for treating children with severe disorders of speech-language production and/or comprehension. Various strategies are used, but research and debate on their efficacy have remained limited to a specific area and have rarely reached the general medical community.

**Objective:**

To systematically evaluate outcomes of AAC interventions in children with limited speech or language skills.

**Methods:**

Searches were conducted (up to December 2012) in the MEDLINE, EMBASE, PsycINFO, CINAHL, DARE, and Cochrane Library databases. Furthermore, relevant journals were searched by hand. References from identified studies were examined. Only RCTs were considered. Trial quality was assessed according to a standardized and validated set of criteria.

**Results:**

Fourteen of 1661 retrieved papers met inclusion criteria. A total of 666 children were included in the review and 7 papers involved only children <5 years old. Papers were of average quality and all but one had been published during the previous 10 years by one of 8 research groups, 5 of which from the United States. Seven studies directly addressed AAC use by children with different disabilities. Seven studies enrolled typically developing children: 5 evaluated the use of AAC technologies by children without disabilities in order to obtain results that could be used to improve interventions in peers with disabilities, and 2 evaluated peers’ attitudes towards children who used AAC. Both interventions and outcome measures varied widely between studies. Overall findings demonstrate the effectiveness of the AAC interventions considered, but the focus on RCTs alone appears too restrictive.

**Conclusions:**

Solid evidence of the positive effects of AAC interventions in children with severe communication disorders must be generated, and different methods are needed besides RCTs. Moreover, it is important that knowledge, research, and debate extend to the medical community in order to ensure clinically effective AAC provision for these children (and their parents).

## Introduction

Ever since non-speech communication systems have been employed in individuals with little or no functional speech, Augmentative and Alternative Communication (AAC) interventions have evolved rapidly. The term AAC includes all forms of communication (other than speech) that are used to express thoughts, needs, wants, and ideas in order to supplement spoken or written communication in individuals with severe disorders of speech-language production and/or comprehension [1].

AAC is only one component of Assistive Technology (AT), which is a broad term referring to assistive, adaptive, and rehabilitative devices that assist an individual in functioning in society at a more appropriate and independent level. AT includes wheelchairs, ramps, and TTYs (phone systems for individuals who are deaf), whereas AAC involves multimodal approaches incorporating gestures, vocalizations, signs, orofacial expressions, as well as picture symbols, voice output devices, or other computer-based technologies, based on what is most successful in meeting the complex communication needs of subjects across different settings. Levels of AAC technology can vary from unaided modes, in which no external device is required (sign languages or gestural cueing systems), to aided AAC [1]. The latter includes low-technology (alphabet boards, symbol-based topic boards, and communication books or programs) and high-technology aided modes (electronics and computer technologies).

The percentage of people who find it difficult to communicate their needs effectively without help is about 1.2% of the general population [2], while approximately 5% of preschool children may have some form of language impairment or delay [3], and less than 0.1% have severe to profound deafness with onset before language is established [4], [5]. While it is generally known that sign language may be a very relevant choice for infants with profound prelinguistic deafness and that its early and full introduction may support development and mental health [6], the existence and relevance of AAC for development in children with communication disorders is less well-known. From 0.3 to 0.6% of children and adolescents may benefit from AAC interventions [7] for a wide variety of communication problems that can be found in association with numerous medical conditions such as autism spectrum disorder, cerebral palsy, intellectual disabilities, and rare genetic syndromes. AAC is described as an important mean to compensate speech, enhance communicative competence, acquire prelinguistic and cognitive skills essential for language development, and facilitate the emergence of speech and language [8]–[13]. Moreover, it is considered very relevant to quality of life by parents and users [14]. The objective of AAC interventions is the long term development of functional communication and, possibly, of language skills. Reducing the communicative gap is, in fact, a critical step, because when a toddler has a severe language and communication delay, his ability to interact socially, gain information, develop his cognitive potential and learn from the environment is significantly compromised, with dramatic consequences on his global development and an increased risk of behavior problems. Demonstrating the efficacy of interventions in AAC has therefore been a central concern in the field for many years [15]–[30]. There has also been debate about which outcomes are to be considered relevant [17], [18], [20], [21] and whether the usual criteria for evaluating evidence may be adequate without modifications [22], [29]. Conducting efficacy research in AAC poses significant challenges because of the paucity and heterogeneity of the population of AAC users, the transactional and dynamic nature of the communication process, the variability of AAC systems and interventions [17], the importance of generalization and maintenance [20], and the key role of communication partners and of social validation of objectives [17], [18], even more so when addressing a pediatric population. AAC candidates may differ significantly in their functioning, even when affected by the same medical diagnosis: intelligence, attention, receptive language, expressive language, adaptive behavior, and motor skills may all be compromised, but to various extents and with different possible combinations. Moreover, communication is a process by which people jointly build meaning [8]. Each partner contributes to the interaction by using language, gestures, eye gaze, body posture, cultural norms, and speech, and the specific characteristics of the communicating partners highly influence the entire process. This, in turn, influences the possible communicative needs of the patient and the shared definition of outcomes [17], [18].

Many journal articles involving AAC have been published during the last decade and the number is increasing. A specific journal (the official journal of the International Society for Augmentative and Alternative Communication) has been published quarterly since 1985 and has been indexed since 2005 by the Institute of Scientific Information (ISI). There has also been a marked growth in the publication of books on the topic. Nonetheless, research and debate seem to have remained limited to a very specific area of rehabilitation and have rarely reached the medical community. A better knowledge of AAC and of its possible impact on children and families would be important for guaranteeing timely referral of children and families and therefore improving their quality of life and helping to prevent behavioral problems and cognitive deterioration. Critical points in AAC research methodology may also be considered as an example of the challenges that need to be faced in building solid evidence for complex interventions with high context involvement in rare diseases.

Efforts to implement evidence-based practice in AAC have been made, also highlighting the importance of properly performed systematic syntheses aimed at determining the effectiveness of interventions in AAC [24]–[28], despite the fact that data are still scant. The number of AAC intervention studies has increased rapidly and a few randomized controlled trials (RCTs) have also been carried out. Although RCTs are the most rigorous way of determining whether a cause-effect relationship exists between intervention and outcome, the appropriateness of such stringent designs has been questioned for the AAC field [22], [29]. Nonetheless, no systematic review of RCTs on outcomes of AAC interventions in children has been carried out, except for one on the effects of AAC on speech production in children with autism. This study which included mostly single-subject experimental design studies and only 2 RCTs, and is now 5 years old [28]. Taking into account the extensive literature on AAC and the range of AAC interventions, a scoping review of RCTs was performed in order to explore the extent of RCT production in AAC, and to help identify target points (population, intervention, comparison, outcomes, and context) for future critical appraisal processes.

## Methods

### Search Strategy

An extensive search of the published literature was conducted. The following electronic databases were searched for articles published in any year up to December 2012: MEDLINE, EMBASE, PsycINFO, CINAHL, DARE, and the Cochrane Library. References from identified studies were examined and relevant journals (EBCAI - *Evidence-based Communication Assessment and Intervention* and AAC - *Augmentative and Alternative Communication*) were hand searched. The general search strategy used was: [(communication aids for disabled OR facilitated communication)] AND (child$ OR adolesce$ OR pediatr$). The search was performed also using the term “alternative and augmentative communication” as free text. The search terms used were specific to each database, according to the Pearl Growing strategy [28]. Reference lists were searched for potentially relevant articles.

### Inclusion Criteria

Criteria for inclusion in the review were: (1) participants aged under 18 years; (2) description of a specific AAC intervention; (3) a comparative group was considered; (4) intervention outcomes were specifically reported; and (5) comparisons were randomized and conducted for intervention and control groups.

Studies were excluded from the review if: (1) participants were aged more than 18 years or insufficient detail was provided to ascertain participant age; (2) outcome assessment was not reported; (3) a specific intervention was not assessed; or (4) the study did not utilize a randomized controlled design.

### Data Extraction and Assessment

All identified abstracts were manually read for their applicability to inclusion and exclusion criteria and potentially relevant articles were obtained and examined. All references retrieved were collected and analyzed using the Reference Manager v.11 program (Institute for Scientific Information, Berkeley, California, USA). Articles meeting inclusion criteria were examined to extract the following information: sample characteristics (age range, clinical characteristics, sample size); experimental and control interventions; outcomes and method used to measure the outcomes, inclusion and exclusion criteria, developmental measures, mean IQ, communication and language measures, and mean communication level at baseline. References of pertinent papers were also scrutinized for additional relevant articles. Study quality of RCTs was assessed using the Delphi list [31].http://adc.bmj.com/content/95/9/717.full - ref-19 This tool includes items relating to whether: randomization was conducted, treatment allocation was blinded, participant groups differed at baseline, and intention to treat analysis was conducted. Total scores are unweighted and ranged from 0 (poor quality) to 9 (high quality). Each study was also assessed using the Jadad point scale [32]. The instrument contains 3 items related directly to reduction of bias (randomized, double-blind, and withdrawals and drop outs), and 7 additional items (in the present evaluation) to check for other markers (objectives, outcome measures, inclusion and exclusion criteria, sample size, interventions, control group, and statistical analysis). Total scores ranged from 0 to 10. Both authors assessed each of 13 pertinent randomized studies independently, and inter-reviewer disagreement was solved by discussion. Agreement on inclusion was calculated using the Kappa statistics.

## Results

### Search Results

**Figure 1:**
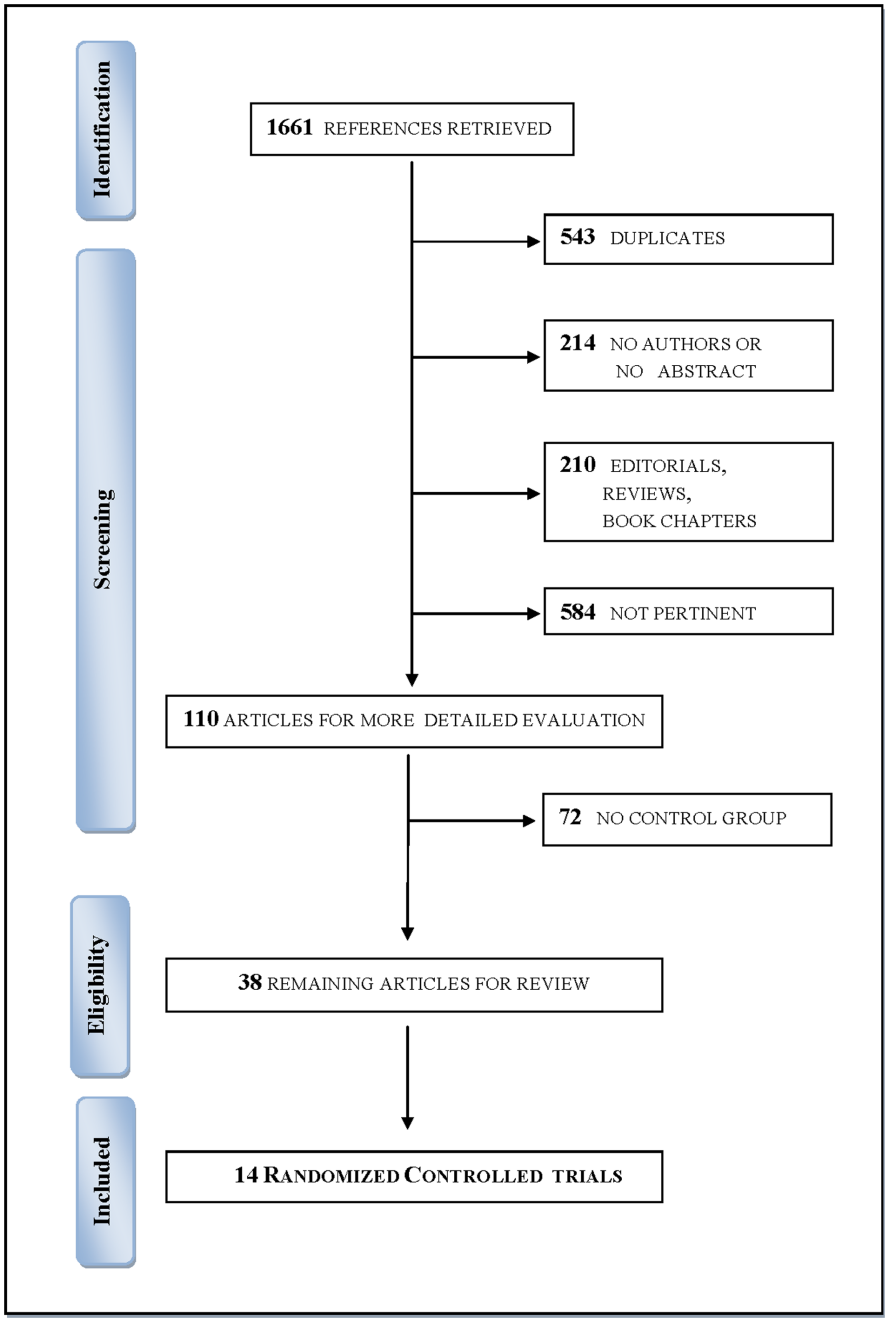
Flow chart of the search strategy used for identification and selection of trials.

The literature search resulted in 1661 titles. A total of 543 duplicates and 1008 non-pertinent or non-appropriate references were deleted, resulting in 110 potentially relevant articles. Authors agreed on 90 of 110 papers (81.8%) selected for reliability check (K = 0.633), and disagreements were resolved by consensus. Thirty-nine studies (35.4%) met inclusion criteria and were controlled studies. Of these, 14 were randomized and were therefore included in the final step of the review (figure 1) [33]–[46].

The 110 pertinent articles were published between 1983 and 2012, with 26 (23.6%) published before 2000. In 2004 the number of papers published increased and the yearly rate remained steady since then. These papers appeared in 50 different journals, 33 of which published only 1 paper each (30%). Only 10 journals published at least 3 papers each, for a total of 45 papers (44.1%). The articles were published by 342 authors, most of whom (76.9%) appeared in 1 paper each, and only 13 of whom (3,8% of all authors) appeared in at least 3 papers. These 13 authors belonged to one of 7 groups (4 American and 1 Australian, 1 Italian, and 1 South African) and produced 32 of the pertinent papers (29.1%). The study by Wu et al. [34] was considered to fall within the scope of AAC and not of deaf education because it was focused on the efficacy of translating icons into written language, a topic that goes well beyond the area of hearing impairment.

### RCTs Identified

Table 1 summarizes the characteristics of the 14 included trials. All are single-center, randomized controlled studies, performed in 3 countries across 3 continents (Africa, Asia, and North America). Eleven of the studies (78.6%) were from USA [33], [35]–[40], [42], [44]–[46] and, specifically, from 5 groups. These groups reported the results (usually as a thorough examination) of the approach they developed. All RCTs were published in the last 10 years, except one published in 1988 [33], and were published in 7 different journals. Six of the journals had an impact factor and the average was 2.64 (1.18–5.01). One journal did not have an impact factor [41].

**Table 1:** Characteristics of the included randomized controlled studies (n = 13) on AAC interventions.

Author(year)country	Age groupin years(mean)	No	Intervention	Comparativegroup	Outcomemeasure	Key outcomefindings	Delphi score	
Studies on children with disability who use AAC								
Yoder &Layton(1988) [33]USA	<9 (5.4)	60	90 individual trainingsessions of 40 minutes(min) each alternatingsign and speech	Children trained withsign alone; with speechalone, or withsimultaneous signand speech	Total number ofdifferent child-initiated spokenwords during thetraining sessions	Training conditionsthat included verbalinput and the expectationof verbal output weresuperior to sign alone infacilitating spontaneousspoken words duringtreatment	5	
Wu et al.(2004) [34]Taiwan	11.0–16.0 (13)	10	Three months trainingwith a Chinese textgenerating computerizedprogram based on apredictive sentencetemplate	Children trainedwith the conventionalteaching method	The literacy aptitudetest and subjectivesatisfactory level	The literacy aptitudetest and subjectivesatisfactory levelimproved significantly(≈40%)	2	
Yoder andStone (2006)[35] USA	1.8–4.5 (2.8)	36(19+17)	Picture ExchangeCommunication System(PECS) 20 minute therapysessions, 3 times per weekfor 6 months (72 sessions),and up to 15 hrs of parenttraining	Responsive Educationand Prelinguistic MilieuTeaching (RPMT)	Rating scale ofcoded variablesduring Early SocialCommunicationScales (ESCS),Unstructured FreePlay with Examiner(UFPE), and parent-child free-playsessions	The RPMT facilitatedthe frequency ofgeneralized turn takingand generalized jointattention more thandid the PECS. However,in children with verylittle joint attentionPECS facilitatedgeneralized requestsmore than RPMT	5	
Yoder andStone (2006)[36] USA	1.8–4.5 (2.8)	36(19+17)	Picture ExchangeCommunication System(PECS) 20 minute therapysessions, 3 times per weekfor 6 months and up to15 hrs of parent training	Responsive Educationand Prelinguistic MilieuTeaching (RPMT)	Systematic analysisof languagetranscripts to countthe frequency ofnonimitative spokenacts in videotapedrecords of examiner-child free playsessions; handcounting of differentnonimitative words,turn taker software tocode objectexchange turns,play coder softwarefor number ofdifferent toystouched.	Children who startedintervention with highobject explorationincreased the numberof nominative wordsfaster with PECS thanwith REPMT. However,the opposite was truefor children whostarted interventionwith low objectexploration.	5	
Yoder andLieberman(2010) [37]USA	1.5–5.0 (3.1)	36	Picture ExchangeCommunication System (PECS) 20 minute therapysessions, 3 times a week for6 months (72 sessions), andup to 15 hrs of parenttraining	Responsive Educationand Prelinguistic MilieuTeaching (RPMT)	Mean n° of pictureexchanges duringESCS-Abridged	The mean n° ofpicture exchangesfor PECS and RPMTgroups were 3.84and 1.01, respectively	3	
Romski et al(2010) [38]USA	1.7–3.3 (2.5)	68	Parent-coached augmentedcommunication input andparent-coached augmentedcommunication output, 24sessions of 30 min each,in three 10 min blocks(play, book reading, andsnack) in which targetvocabulary was used. 18sessions were in laboratorysetting and 6 in child home,distributed over a medianof 15–16 wks	Parent-coached spokencommunicationintervention	Augmented wordsand spoken wordsuse; communicationinteraction skills	Vocabulary size wassubstantially larger forAAC interventionsthan for spokencommunicationintervention	4	
Romski et al.(2011) [39]USA	1.6–3.3Children(2.5)31–45Parents(37)	53	Parent-coached augmentedcommunication input andparent-coached augmentedcommunication output, 24sessions of 30 min each,in three 10 min blocks(play, book reading, andsnack) in which targetvocabulary was used. 18sessions were in laboratorysetting and 6 in child home,distributed over a medianof 15–16 wks	Parent-coached spokencommunicationintervention	The 20 items of theParent perception oflanguagedevelopment(PPOLD)	More positiveperceptions ofsuccess after allinterventions.Perceptions of theseverity of the child’slanguage difficultiesdecreased for AACinterventions, butincreased for thespoken intervention.	4	
Studies on typically developing children								
Drager et al.(2004) [40]USA	3.0–3.9 (3.5)	30	Use of dynamic displaywith 61 vocabulary items,in a contextual sceneformat 4 learning sessions(30 min each) and 1generalization session	Participants in the grid-single-symbol and grid-single shot menu condition	The children’saccuracy in locatingtarget vocabulary	Children performedsignificantly betterwith AACtechnologies in acontextual scene formatthan in a grid format,but by the fourthsession the differencewas no longersignificant	4	
Basson andAlant(2005) [41]South Africa	6.0–6.9 (6.4)	46	Exposure and one sessiontraining with a thematically-organized communicationoverlay with 16 PictureCommunicationSymbols ™ (PCS)	Children receivingexposure only and notraining	The accuracy ofchildren’s selectionof the symbol inresponse to itsspoken label,representing thesymbol’sguessability	The 16 PCS symbolshad an iconicity of12.5–25% and therewas a significantimprovement in thesecond session,greater in the trainedgroup	3	
McCarthyet al. (2006)[42] USA	2.3–2.9 (2.7)	20	3 exposure sessions, 10–30 min each, toredesigned enhancedscanning technique toreduce learning demands	Traditional scanningtechnique	The child’s accuracyin selecting targetitems	The children inenhanced scanningcondition were morethan twice as accuratein their scanningperformance as theirpeers in the traditionalscanning condition	4	
Alant et al.(2010)[43]South Africa	5.1–6.9 (5.8)	60	Sequential exposure todifferent types of coloredmeaningful symbols	Sequential exposure todifferent types ofcolored arbitrary formsof 3 color conditions	The accuracy andrate with which theparticipantsidentified the itemsin a stimuli array	The sequential exposures(orderings) impactedboth on time andaccuracy for meaningfulsymbols and arbitraryforms, within specificinstances	4	
Schlosseret al.(2012) [44]USA	3–3.10(16 children)3.11–4.9(18 children)4.10–5.8(18 children)(4.3)	52	Animated representationof 24 verbs and 8spatial prepositions fromthe ALP AnimatedGraphics Set	Static representation of24 verbs and 8 spatialprepositions from theALP Animated GraphicsSet	Effect of symbolformat (animated,static), of word class(verb, preposition),and of age (3, 4,5 yrs old) ontransparency, nameagreement, andidentification	Animation effect wassignificant fortransparency, but notfor name agreementand identification.The effect was morepronounced for verbsthan prepositions, andolder childrenoutperformed youngerchildren	**4**	
Studies on peer attitudes towards children who use AAC								
Beck et al.(2003) [45]USA	7–8(30 children)9–10(31 children)11–12(34 children)(9.6)	95	A school-basededucational programproviding informationand a 16 min videoregarding AAC, incombination with arole-play experience	Children receivinginformation and videoalone	The 26 items of theAssessment ofAttitudes TowardAugmentative andAlternativeCommunication(AATAAC) scale	A greater positiveeffect of the informationplus role-play experiencecompared to the effectsof being giveninformation alone forolder children and boys	4	
Beck et al.(2010) [46]USA	14 yrs(26 adolescents)15 yrs(42 adolescents)16 yrs(40 adolescents)17 yrs(20 adolescents)18 yrs(8 adolescents)(15.6)	136	8 videotapes depicting 4different gendercombinations of AACusers and communicationpartners, a dynamic touchscreen device	AAC users with a statictouch screen	The 33 items of theAssessment ofAttitudes TowardAugmentative andAlternativeCommunication-2(AATAAC-2) scale	Type of AAC devicecombined withfamiliarity with peoplewith disability andgender contribute toadolescents’ attitudetowards people whouse AAC	5	

#### Participants

Seven studies regarded children with disabilities [33]–[39] and seven involved typically developing children [40]–[46]. The latter were, nonetheless, included in the review because they evaluated ways to improve or support interventions in peers with disabilities. One of the studies on children with disabilities regarded profoundly deaf children whose associated disabilities were not described [34]. As mentioned before, this study was included because it involves research on augmentative communication technologies to translate icons into written language. In the studies regarding children with disabilities, the number of children involved ranged from 10 to 68 (median 36), with a total of 299 children included. Three studies referred to the same patients [35]–[37]. The ages of the 227 children ranged from 1.5 to 16 years, with a mean of 4.6. Five trials involved only children (157/227; 69.2%) less than 5 years old [35]–[39]. Six studies [33], [35]–[39] reported both developmental level/IQ at baseline (mean DQ/IQ between 40 and 60) and communicative level (mean expressive language between 9.9 and 21.5 months; mean receptive language between 14.1 and 20 months). One of the studies [34] did not report the developmental/cognitive level of the children enrolled nor their communicative competence at baseline, but reported literacy level (mean 73) because the study described an intervention aimed at improving literacy in deaf children. Table 2 reports additional details on participant characteristics.

**Table 2:** Group designs of randomized controlled trials involving children with disability who use AAC.

Author(year)country	Inclusioncriteria	Exclusioncriteria	Developmentalmeasures	Mean IQ (SD)at baseline	Communicationand languagemeasures	Meancommunicationlevel (SD) atbaseline
Yoder &Layton(1988) [33]USA	−<9 yrs old	-hearing orvision deficits	Leiter or Bayleyor Merril-Palmer	Mean nonverbal IQ42.9 (17.8); 40.5(33.1); 41 (23.8);44.4 (24.4)	Expressive andreceptive scalesof SequencedInventory ofCommunicationDevelopment(SCID);	Mean receptivelanguage 17.3(6.7); 14.1 (4.2);14.9 (5.9); 16.2(4.1)
	-Autism or PDD-NOS: CARS scorebetween moderateand severe				parentquestionnaireof initialexpressivevocabulary;	Expressive language15.2 (12.6); 9.9(6.9); 12.3 (6.5);11.7 (8.8)
	-expressive andreceptive age <28months (mths) onSICD				experimenterdesignedinstrument forelicited verbalimitation	Initial vocabulary6.8 (7.3); 4.8 (7.1);6.5 (7.6); 3.9 (7.9)
	-expressive vocabulary<25 words on parentquestionnaire					Elicited verbalimitation 298.1(342.2); 281.5(321); 258.6(348.1); 287.7(360.7)
Wu et al.(2004)[34]Taiwan	-profoundly deafstudents	- not reachingprerequisiteliteracy level	not reported	not reported	Literacyaptitude test	Mean literacy level:73 (8.12); 73 (5.7)
	-fifth grade					
	-primary deafschoolin Taiwan				Semanticintegrationindex perutterance	Mean Semanticintegration level:0.42 (0.10); 0.41(0.13)
Yoder andStone(2006) [35]USA	-Autism or PDD-NOS at ADOS	-severe sensoryor motor deficits	Mullen scalesof early learning(MSEL)	Mean MSELcomposite scoreof 55 (7) for PECSand 54 (6) forRPMT (childrenunder 49 excluded)	Mullen scalesof early learning(MSEL). McArthurCommunicativeDevelopmentInventories(CDI), EarlySocialCommunicationScales (ESCS),unstructured freeplay with examiner (UFPE)	CDI: mean wordsunderstood 108(87) for PECS and62 (49) for RPMTESCS and UFPE:
	−18–60 mths	-English notprimary languageat home				- mean n° of**children** initiatingjoint attention: 3 (4)in PECS and 2 (2)in RPMT;
	−<10 words					- mean n° of objectsexchanges: 5 (5) forPECS and 2 (3) forRPMT
	-hearing screening ok					
Yoder andStone(2006) [36]USA	-Autism or PDD-NOS at ADOS	-severe sensoryor motor deficits	Mullen scalesof early learning(MSEL)	Mean MSELcomposite scoreof 51 (5,3)(children under49 wereassigned 48)	Mullen scalesof early learning(MSEL).	SFPE: - mean n° ofnonimitative spokenacts 0.25 (0.84)
	−18–60 mths	-English notprimary languageat home			Fifteen minutesemistructuredfree play withexaminer (SFPE).DevelopmentalplayassessmentTurntaking procedure	- mean n° ofdifferentnonimitative word0.17 (0.56)s
	−<20 words					-mean n° ofcommunication acts8.4 (10.5)
	-hearing screening ok					
Yoder andLieberman(2010) [37]USA	-Autism or PDD-NOS at ADOS	-severe sensoryor motordeficits	Mullen scalesof early learning(MSEL)	50.32 (5.2)PECS, 51.76(5.41) RPMT	Mullen scalesof early learning(MSEL). McArthurCommunicativeDevelopmentInventories(CDI), EarlySocialCommunicationScales (ESCS),unstructured freeplay withexaminer (UFPE)	Mullen expressivelanguage score:19.47 (1.26 PECS,21.59 (3.36) RPMT
	−18–60 mths	-English notprimary languageat home				Mullen receptivelanguage score:19.26 (0.45) PECS,19.41 (0.51) RPMT
	-<10 words					
	-hearing screening ok					
Romski et al.(2010) [38]USA	−24–36 mths	-autism	Mullen scalesof early learning(MSEL)	Mean MSELcomposite scoreof 60 for AC-Iand 59 for AC-Oand SC	MSELexpressive andreceptive scales,McArthurCommunicativeDevelopmentInventories,SequencedInventory ofCommunicationDevelopmentand ClinicalAssessment ofLanguageComprehension	Receptive language18 mths;20 mths; 19 mths
	−<10 intelligiblespoken words	-deafness/hearingimpairment				Expressivelanguage 12 mths;13 mths; 13 mths
	-score of less than12 mth onexpressive languagescale of MSEL	-delayed speechand languageimpairment				
	-at least primitivecommunicationabilities					
	-motor skills thatpermitted the childto touch the symbols					
	- English as primarylanguage at home					
Romski et al.(2011) [39]USA	As in previous study	As in previousstudy	Mullen scalesof early learning(MSEL)	Mean MSELcomposite scoreof 60 for AC-Iand 59 for AC- Oand SC	MSELexpressive andreceptive scales,McArthurCommunicativeDevelopmentInventories,SequencedInventory ofCommunicationDevelopmentand ClinicalAssessment ofLanguageComprehension	Receptive language18 mths; 20 mths;19 mths
						Expressive language12 mths; 13 mths;13 mths

Five studies [40]–[44] involving typically developing children regarded ways of improving the learn ability of AAC systems, and involved between 20 and 60 children (median 46), with a total of 208 children included. The children’s ages ranged from 2.3 to 6.9 years (mean 4.9). Two trials involved only children less than 5 years old (50/208 children; 24%) [40], [42].

The other 2 studies [45]–[46] regarded peer attitudes towards AAC users, and the number of children involved ranged from 95 to 136 (median 115), with a total of 231 children included. The children’s ages ranged from 7 to 18 years (mean 13.1).

Children with a history of developmental delay, learning, hearing, or uncorrected vision problems, or in whom the local language (English or Africaans) was not the mother-tongue of the child, were excluded from all 7 studies involving typically developing children.

#### Study-Quality Assessment

The Delphi score ranged from 2 to 5 (mean 4.0), with 9 as the maximum possible score. No studies concealed the treatment allocation or completely blinded the outcome assessor, the care provider, or the patient.

The Jadad score ranged from 3 to 8 (mean 5.1), with 10 as the maximum score possible. No studies were described as double-blind, and only one justified the sample size [44]. Only 1 study described withdrawals and drop outs [41] and 8 the statistical analysis methods used [33], [35]–[37], [40], [41], [44]–[46].

#### Outcome assessment

The outcome measures used in the 14 studies differed widely. Of the 7 studies [33]–[39] in which efficacy of AAC interventions was tested in children with disabilities, one involved joint attention during communication, object exchange turns and requests [35], and the generalization of use of symbols [37], one regarded the increase in target vocabulary and communicative interactions [38], one the improvement in reading comprehension [34], and one the parents’ attitudes following intervention [39]. Four studies’ outcomes included speech directly (number of spoken words during treatment [33], number of different child-initiated spoken words during the training sessions [35], number of non-imitative spoken acts and different words [36], or increase in target vocabulary [38]). In 5 studies [40]–[44] AAC technologies were used in typically developing children without disabilities, in order to compare different training levels and types of tools used or of interventions performed, and to use results to improve interventions in peers with disability. In 2 studies [45], [46] outcome measures were focused on the attitudes of typically developing children towards peers who use AAC, as a possible relevant aspect of communication partners’ interaction with users.

Details of group designs of the randomized controlled trials involving children with disabilities are reported in Table 2.

Yoder & Layton [33] tested the main, and interaction, effects of 4 different training conditions (alternating presentation of sign and speech, sign alone, speech alone, and simultaneous presentation of sign and speech) and of pretreatment elicited verbal imitation ability in predicting child-initiated spoken language use during training sessions of minimally verbal autistic children less than 9 years old. Training conditions that included verbal input and the expectation of verbal output were superior to sign alone in facilitating spontaneous spoken words during treatment, and pretreatment verbal imitation ability positively predicted the size of the child-initiated spoken vocabulary. Exploratory analysis indicated that pretreatment age and IQ may also predict spoken language development during training.

The Wu et al. study [34] proposed a computerized, graphic interface based on a predictive sentence template tree for translating icons of Taiwanese sign language into Chinese written sentences, and compared it with a conventional teaching method in children with profound hearing impairment in the fifth grade of a primary school for the deaf in Taiwan. Findings showed an improvement rate in Chinese reading comprehension in deaf children in the intervention group. The proposed system applies the design methodology of sentence prediction and construction to develop the task or domain-specific sentence types.

The study by Yoder & Stone [35] compared the relative efficacy of two communication interventions, Responsive Education and Prelinguistic Milieu Teaching (REPMT), and Picture Exchange Communication System (PECS), on initiating joint attention, on object exchange turns, and on requests, in 36 preschoolers 18–60 months of age with autism spectrum disorders and less than 10 spoken words. In autistic children with some joint attention, REPMT facilitated the frequency of generalized turn taking more than PECS, while the opposite occurred in children who began the study with no joint attention.

The second article by Yoder & Stone [36] regarded the same research, but considered spoken communication acts as outcomes. The growth rate of different, spoken, nominative words was faster in the PECS group than in the REPMT group for children who began treatment with relatively high object exploration, while the opposite occurred for children who began treatment with low object exploration.

The Yoder and Lieberman [37] study, by the same authors [35], [36], represented an extension of the previous studies, focusing on the generalization of use of symbols. The study found that young children with autism who received PECS training increased the number of picture exchanges to a greater extent than children receiving REPMT, when in a controlled context that was different from the training context in several dimensions. PECS use may thus be one way to help a child not only to begin to use joint attention towards objects and people, but also to use it to communicate in generalized contexts.

Romski et al [38] compared three parent-coached language interventions (augmented communication input and output and spoken language intervention) in young children 24–36 months old with developmental delays who began with fewer than 10 spoken words, and found that augmented language interventions increased target vocabulary and communicative interactions to a greater extent than spoken communication interventions. The authors concluded that AAC does not hinder, but actually aids, speech production abilities in young children with developmental delay, and does so even over a short period of time. They state that more research is needed on the interaction between comprehension and production of augmented and spoken words, and that this interaction appears to be more complex than was initially hypothesized.

Finally, the study by Romski et al [39] focused on parental perceptions of language development in toddlers from the previous study [38], demonstrating that augmented language intervention also has a positive impact on parental perception of language development in their children. Both studies highlight the important role AAC interventions can play at a very early age for children who are having difficulties with speech and language development.

The Drager et al. study [40] investigated the learning demands of different AAC dynamic displays in typically developing 3 year old children. Results showed that, initially, transparency was poor for all AAC technologies used, but participants performed better across successive sessions. By the second learning session, children in the contextual scene-screen shot condition performed significantly better than children in the two grid conditions, but by the fourth session the difference was no longer significant. Embedding language concepts within contextual scenes may be an effective approach for young children learning dynamic display AAC technologies. However, authors conclude that the systems differed by more than one characteristic and the performance of typically developing children may not be fully generalizable to that of older children or children with disabilities. Moreover, functional use in free play remained low, confirming the importance of better exploring the different effects of support provided in order to facilitate learning, generalization, and spontaneous use.

The Basson and Alant study [41] set out to determine how accurately typically developing, 6 year old, urban, Africaans speaking children who had at been enrolled for at least 6 months in preschool could identify 16 Picture Communication Symbols (PCS), with and without training. Results confirmed that a rather low percentage of symbols can be correctly identified on first exposure based only on iconicity. A significant improvement at retest, although greater in the intervention group, was seen in both experimental and control groups, showing that iconicity may be only one of the components that facilitate the learning and memory of AAC symbols, and that exposure and training also play a relevant role. The number of participants and of symbols considered was limited, and again performance of typically developing children may not be fully generalizable to that of children with disabilities. The authors therefore conclude that different symbols, different grid sizes, different ages and cultural groups, and different training strategies need to be considered in future studies.

The purpose of the McCarthy et al. study^42^ was to investigate the learning demands of a redesigned scanning technique and to compare it with traditional scanning in typically developing 2 year olds. Results indicate that, after three learning sessions, most typically developing 2 year olds increase their accuracy with the redesigned scanning technique further than with traditional scanning. However, results may not be generalizable to children with disabilities, and other scanning designs and the development of new and innovative access techniques need to be investigated.

The Alant et al. study [43] examined the role of color on rate and accuracy in identifying symbols in typically developing children. Findings indicate that the use of different colored symbols in sequential exposures impacts the time and accuracy of symbol location, and contributes to understanding how typically developing children locate different types of symbols in a context in which the color of symbols changes. The findings confirm both the complexity of factors affecting visual search and processing and the fact that understanding visual search processes requires a sound analysis of the multiple factors embedded in the process within a specific task or context.

In the study by Schlosser [44] et al, the effect of animation on transparency, name agreement, and identification of graphic symbols for verbs and prepositions was evaluated in typically developing preschoolers of 3 age groups. The animation effect was significant for transparency, but not for name agreement or identification. The effect was more pronounced for verbs than prepositions. A developmental effect was observed for each measure. The authors suggested that there is a need to replicate the study with different symbol sets, with child directed control of animation, and with additional symbols on the display. In the Beck et al. study [45] typically developing children in 2nd, 4th, and 6th grade of a small suburban elementary school with no children with disabilities in their class were given one information session on peers using AAC, alone or combined with role playing, in order to evaluate possible changes in their attitude towards these peers. In the group of older children and, particularly, in boys, the association of a role-playing experience resulted in higher positive self-reported attitude scores toward peers who use AAC than did the provision of information alone. The authors conclude that, even though a change in attitude does not necessarily imply a change in behavior, determinants of children’s attitudes towards their peers who use AAC and of formation of friendships between them need to be explored further.

The second study from Beck et al [46] is similar to the previous one [45] and is aimed at investigating elements of high school students’ self-reported attitudes towards peers who use AAC. The study found that the type of AAC device, along with familiarity with people with disabilities and gender, contribute to adolescents’ attitudes towards people who use AAC.

## Discussion

To our knowledge, this is the first scoping review to investigate outcomes of AAC interventions that focuses only on RCTs and uses a standardized set of criteria for the assessment of the methodological quality and strength of evidence of retrieved RCTs studies. Previous reviews also considered other study designs, such as non randomized group studies [23]–[25] or single case experimental designs [20], [24]–[26], [28], and therefore used a broader approach for selecting papers for the review [47].

The results of the retrieved studies, while providing some information on the effects of AAC interventions, confirm numerous limitations in the use of RCTs to evaluate AAC interventions:

- all trials were described as randomized, but the risk of bias was unclear in the majority of studies because the methods of random-sequence generation and allocation concealment were not explicitly reported;
- non-uniform formal reporting of outcome results reduced the power of findings and their communication to readers;
- the comparison groups used in the reviewed studies differed in the criteria employed, both within and between studies, potentially causing bias because no concealed randomization was used;
- none of the included studies used a random selection strategy or a case-controlled design, so the quality score could not exceed 5, despite the highest potential score of 9;
- all included studies had relatively small sample sizes, especially 4 of the studies enrolling patients with disabilities (10 in one study and 36 in the other three) [34]–[37]. Only three studies [33], [38], [39] on children with disabilities enrolled a slightly greater sample (60, 68, and 53 children): the two most recent were by Romski et al. [38], [39]. The larger samples regarded the two studies by Beck et al [45], [46] (95 and 136 children, respectively), in both cases regarding typically developing children’s attitudes towards peers using AAC. Comparing such relatively small groups might also be a potential cause of bias;
- most of the reviewed studies explored different AAC techniques or even single components of the technique, making comparison between studies very difficult;
- the outcomes evaluated differed between studies and also within individual groups [35]–[37].

Because the entire group of retrieved RCTs was characterized by entirely different study outcomes, no attempt was made to aggregate these outcomes across studies. Similarly, effect size estimation was not used since studies differed substantially in design features and quality.

AAC intervention is a long term, complex, multimodal process that needs to be incorporated into daily life. It includes prescription, development, and customization of AAC systems to meet the unique needs of each user; instructions for the individual who uses AAC in various linguistic, operational, social, and/or strategic skills following various instructional protocols; instructions for facilitators in interaction strategies to reduce opportunity barriers and support effective communication; and instructions for facilitators in the operation, maintenance, and ongoing development of the AAC systems used [8], [20], [23]. Furthermore, each one of these intervention components involves multiple procedures. Most of the published studies were focused on separate effects of single components of AAC intervention, while the intervention itself is, in fact, a multidimensional process whose ultimate effect may be quite different from the sum of its components. In this context, group designs are difficult to implement because of the small AAC population and the wide variability within it. Children have complex communication disorders, arising from different medical diagnoses, which may lead to differing disabilities. Enrolled populations range in age from infancy to late teens, and vary widely in functional profiles such as movement, cognition, communication, receptive and expressive language, learning characteristics, vision, and hearing. They also vary in their educational setting (mainstream schools or special education), previous and concurrent interventions, and concurrent medical conditions. In addition, children will experience different social relationships and interact with many different people in many different environments. Each of these factors will influence communication and interventions, especially since communication is a process by which people build shared meaning. Correcting for the effect of these variables in RCTs is extremely difficult. Moreover, AAC intervention increases the complexity of human interaction and acts on several specific domains. The effects of intervention may therefore have an impact on a wide variety of behaviors, and outcomes in one domain may influence outcomes in other domains without the possibility of separating out the effects. In the UK Medical Research Council’s (MRC) definition [48], interventions are considered to be complex when there is a high number of components and interactions within the experimental and control environments, in the number or difficulties of behaviors required by those delivering or receiving the intervention, in the number of groups or organizational levels targeted by the intervention, in the number and variability of outcomes, or in the degree of flexibility or tailoring of the intervention permitted, and AAC interventions fully fit the definition.

Due to the above limitations, it has been argued that RCTs are not first line in complex interventions [48] and that they are possibly not appropriate for AAC research involving individuals with disabilities [17], [19]–[22]. Results of the present systematic review on RCTs seem to confirm these authors’ conclusions. Some of the critical points in obtaining adequate evidence in AAC have, in fact, already been analyzed by various authors, and solutions suggested [16]–[23], [27]–[30], but these have somehow remained confined to specific journals and the debate has not reached the general medical literature. The single subject experimental design (SSED) is considered to be a relevant design option in AAC [28], and is, in fact, widely used in the field. SSED considers each subject as his-her own control, and methodologies for analyzing, in detail, the quality of SSED and for synthesizing the results of various studies through meta-analysis have been developed for AAC [22], [29], and a different hierarchy of evidence has been proposed [22]. However, other alternatives should also be considered, since the quasi-experimental research design could be an appropriate approach and should be tested in the AAC field [19]. In particular, the most commonly used design, the nonequivalent groups design, which substitutes statistical “controls” for the physical control of the experimental situation through a pre-test/post design, should be used. The design is the same as the classic controlled experimental design except that the subjects cannot be randomly assigned to either the experimental or the control group, and the researcher cannot control which group will get the treatment. Participants do not all have the same chance of being in the control or the experimental groups, or of receiving or not receiving the treatment. Such a design could potentially be a better adjusted approach to evaluate the efficacy of interventions in children with complex disabilities.

Randomized studies remain the gold standard when enrolling typically developing children, involving either ways of improving the learnability of systems in order to make them more developmentally sensible or interventions aimed at modifying peer attitudes towards AAC users. A significant number of subjects can be more easily reached, and randomization can be employed, when addressing these populations, and the use of non disabled subjects in AAC research has therefore been widely discussed [48]–[50]. Typically developing children may be enrolled to evaluate symbol acquisition, interaction abilities, selection techniques, speech generating device usability, perception of communicative competence, and language acquisition. Nonetheless, the RCTs retrieved regarded only symbol acquisition, speech generating device usability, and peer attitudes. In the first two situations, the main question concerns external validity: the performance of typically developing children can provide interesting suggestions for future research and development, but it may not be fully generalizable to a population of people who are communicatively impaired. Moreover, the outcomes analyzed in the studies retrieved appear to be partial and very limited when compared to the outcomes expected from AAC interventions. Learnability of a few isolated symbols over a very short period of time is, in fact, very different from using hundreds of various symbols in the long term and in fully functional, everyday communication exchanges, and probably implies very different underlying mechanisms and motivation.

The third situation could be more promising for future research development. Up to now, the majority of the research on interventions has focused on evaluating modifications in the behaviors of AAC users rather than in their conversational partners, while it is known that partners play a key role not only in communication skill acquisition, but also in generalization and maintenance [21]. Studies on the training of conversation partners could reach larger numbers of subjects (each child has many conversation partners) and could more easily be randomized. Larger numbers would also permit an analysis of the effects of different components, namely the impact of having an adult or a child as a partner, the different relationships influencing children (parents, siblings, grandparents, other family members, teachers, classmates, etc), and the role of gender, educational level, previous training, and present communication style. Nonetheless, as found by the studies included in this review, RCTs are very limited and not of optimal quality, making this an area of research that needs significant effort.

Blinding appears to be another relevant point in AAC research. While blinding of the subject, the family, and the interventionist is next to impossible, blinding of the assessor is generally feasible and should be pursued and reported.

Given the extreme variety of subjects that are candidates for an AAC intervention, as much detailed information as possible should be provided regarding the above mentioned patient characteristics (age, cognitive level, receptive and expressive language, attention, behavioral phenotype, previous AAC experience, type of school placement, full diagnosis including comorbidity, etc) in order to determine in whom the intervention was successful and to permit replication of findings. Most tests that explore neuropsychological functions have been created and standardized for typically developing children, and require the integrity and integration of other functions in addition to the one evaluated. This may lead to an underestimation of the extent to which functioning is not homogeneous between children, particularly when considering subjects with severe motor or intellectual disabilities. The homogeneous reporting of subject characteristics and the identification of more appropriate and shared instruments for evaluation [53] are very relevant topics for future evolution [19], [50]–[52].

AAC research has many interesting components. Communication is one of the fundamental human rights, and its impairment results in significant consequences in various areas of child development. Lack of functional communication is generally a life-long condition that severely impacts quality of life of subjects and their families, and is highly correlated with subsequent behavioral problems and high social and economic costs. Access to AAC interventions is still an unmet need in most countries: the few studies available [2], [4], [54]–[56] report from 22 to 60% of children not receiving any AAC intervention, depending on the years considered and on the geographical area. The main barriers identified are resource availability issues (lack of funding, limited access to AAC equipment, etc) and lack of training, and time available, of professionals. Service development and access, as well as the set up of complex interventions and their evaluation appears to be more critical in developing countries and in non English-speaking countries due to linguistic, cultural, and socioeconomic reasons. The results of this review confirm that interest in, and willingness to face, the challenge of evaluating interventions is still limited to a few research groups (mainly American), with a long-standing experience in the matter, suggesting that production and acquisition of “evidence” in the field needs further effort and participation. Time and resources are needed for guaranteeing all children in need of ACC interventions efficacy-proven, accessible techniques, since up to now these needs have remained neglected for the majority of patients.

None of the retrieved RCTs were multicenter studies, suggesting that intergroup collaboration is difficult in the area, also due to the different complexities of AAC patients and, possibly, to linguistic differences between countries. However, collaborative studies are efficacious approaches that favor the transferability of acquired knowledge into common practice, overcoming difficulties and converging on intervention choices. This type of study therefore represents an achievement in the AAC field.

AAC intervention evaluation also represents an interesting example of complexity [48], with similarities to research in rare diseases. The identification of valid and reliable outcome measures appears critical. Possible changes are, in fact, multidimensional, and in order to be measured they need different tools in different domains (language comprehension, symbol and language use, functional communication, cognitive development and learning, participation and inclusion, quality of life, decrease in negative behavior, child and family satisfaction, etc) and require more long term evaluations. The interactive nature of the communication process makes the participation of users, family members, facilitators, teachers, and professionals particularly important in defining objectives. Different people of different ages and with different roles, however, may lead to different outcomes, so social validation techniques need to be included from the start, when the research question is defined, and not at the end of studies. This could be an issue of significant relevance in research in the field of complexity [17], [18], [48]. As in most complex interventions, an improvement in transparency and quality of reporting is also a very relevant topic for future development [17], [24], [48], [57] for all study designs. A better understanding of the different contexts in which an intervention is applied, as well as of the different possible ways of implementing it that can preserve intervention integrity, is essential [58], [59], as is the clear description of the intervention theory base, modeling of components, outcomes, pilot testing, and process of evaluation alongside the clinical trial. Criteria proposed by Mohler et al. [60] appear interesting and consistent both with the methods of development recommended by the EQUATOR network and with previous discussions on the topic in the AAC field.

## Conclusions

Solid evidence of the positive effects of AAC interventions in children with severe communication disorders still needs to be generated. The efficacy of interventions in AAC remains a central concern because of the scant evidence, and the debate has mostly remained limited to specialized literature and has not reached the general medical field. Efficacy research in AAC poses significant challenges due to the paucity and heterogeneity of the population of AAC users, the transactional and dynamic nature of the communication process, the variability of AAC systems and interventions, the importance of generalization and maintenance, the key role of communication partners and of social validation of objectives, and the impact of different languages and cultures on the transferability of results. The low quality of the randomized controlled studies analyzed in this review confirm both the complexity of evidence-building in this field and the fact that studies based on different methodologies are needed in addition to RCTs. No evidence of any harmful effects of AAC in children with speech and language difficulties and their families has, however, been found, and positive trends in communication were shown. With access to appropriate assistive technology at the early stages of development, young children with complex communication needs may be able to maximize their language and communication development and achieve their full potential. Additional research (collaborative and multicenter), designed in innovative ways that can address the complex, multifactorial aspects of the field, as well as studies of higher methodological quality, are therefore urgently needed.

Moreover, it is important that knowledge, research, and debate extend to the medical community in order to ensure clinically effective AAC provision for these children (and their parents).

## Supporting Information

Checklist S1 **PRISMA Checklist.**
(DOCX)
